# Biomechanical performance evaluation of a modified proximal humerus locking plate for distal humerus shaft fracture using finite element analysis

**DOI:** 10.1038/s41598-023-43183-x

**Published:** 2023-09-27

**Authors:** Jung-Soo Lee, Kwang Gi Kim, Yong-Cheol Yoon

**Affiliations:** 1 Department of Health Sciences and Technology, Gachon Advanced Institute for Health Sciences and Technology (GAIHST), Gachon University, Incheon, 21999 Republic of Korea; 2 Department of Biomedical Engineering, College of Medicine, Gachon University, Incheon, 21565 Republic of Korea; 3 Medical Devices R&D Center, Gachon University Gil Medical Center, Incheon, 21565 Republic of Korea; 4 Orthopedic Trauma Division, Trauma Center, Gachon University Gil Medical Center, Incheon, 21565 Republic of Korea

**Keywords:** Trauma, Biomedical engineering

## Abstract

The extra-articular distal humerus plate (EADHP) has been widely used for surgical treatment of distal humerus shaft fracture (DHSF). However, the surgical approach, fixation methods, and implant positions of the EADHP remain controversial owing to iatrogenic radial nerve injury and complaints such as skin irritation related to the plate. Anterior plating with a modified (upside-down application) proximal humerus locking plate (PHILOS) has been proposed as an alternative, However, research on its biomechanical performance remain insufficient and were mostly based on retrospective studies. This study quantitatively compared and evaluated the biomechanical performance between posterior plating with the EADHP and anterior plating with a modified PHILOS using finite element analysis (FEA). The FEA simulation results that both the EADHP and PHILOS had adequate biomechanical performance and stability under axial, bending, and varus force load conditions. The PHILOS has a fixed stability comparable to that of the EADHP, and fixation was achieved using only four locking screws within a fixed range of 30 mm just above the olecranon fossa. The results show that the PHILOS could be an option for the fixation of a DHSF when considering the dissection range and complaints (e.g. skin irritation) associated with the EADHP.

## Introduction

Humerus fractures account for approximately 5 to 8% of all fractures^1^, and 11 to 35% of humerus shaft fractures are located at the distal third of the humerus^2,3^. Distal humerus shaft fracture (DHSF) requires surgical treatment rather than conservative treatment with functional bracing owing to its adjacency to the elbow joint, difficulty in controlling distal humerus fragments, and elbow stiffness caused by long-term immobilization^4,5^.

However, the surgical treatment of DHSF remains a challenge because anatomically, the transition area changes from concave or convex to flat, and the lateral length of the distal humerus is narrow with limited space for the plate and screws^6,7^. Biomechanically, the displacement of distal humerus fragments is severe because of the various muscular forces acting on the fracture^8^. Clinically, reduction and fixation are difficult because of the small distal humerus fragments, and the possibility of radial nerve injury due to their proximity to the nerve^6^. Various surgical approach, fixation methods, and implant positions have been developed to deal with these challenges^9–15^ but remain controversial.

Posterior plating with extra-articular distal humerus plate (EADHP; DePuy Synthes Inc.) fixation is widely used to treat DHSF, and with its “J”-shaped titanium plate precontoured design^16–20^, the EADHP has the advantages of direct visualization of the radial nerve and sufficient fixation of the distal^3,20,21^. However, there is a risk of iatrogenic radial nerve injury during dissection or plate fixation and removal^16–19,22^, skin irritation due to plate protrusion, and limited range of motion of the elbow joint^17,19,23^.

Anterior plating via an anterolateral approach has been proposed as an alternative to deal with these issues. However, concerns have been raised about unstable fixation because of fixation directly above the coronoid fossa. Lim et al.^18^ reported that anterior plating with a modified proximal humerus locking plate (PHILOS; DePuy Synthes Inc.) exhibited a fixation and biomechanical performance comparable to that of posterior plating with the EADHP in bending, torsion, and compression force. Sohn and Shin^22^ suggested anterior plating with a modified PHILOS as an alternative treatment for a DHSF. To the best of our knowledge, no research has been conducted on how small distal fragments can be fixed by anterior plating in DHSF, and the number of screws that should be fixed.

In this study, a fracture model of a DHSF was constructed, which was fixed by posterior plating with the EADHP and anterior plating with a modified (upside-down application) PHILOS. The stress distribution was simulated by finite element analysis (FEA). The purpose of this study is to quantitatively compare and evaluate the biomechanical performance between posterior plating with the EADHP and anterior plating with the modified PHILOS, which are representative fixation methods in DHFS through an FEA study. We attempt to find out the fixation range and state of anterior plating with the modified PHILOS.

## Material and methods

### Data preparation

**Figure 1:**
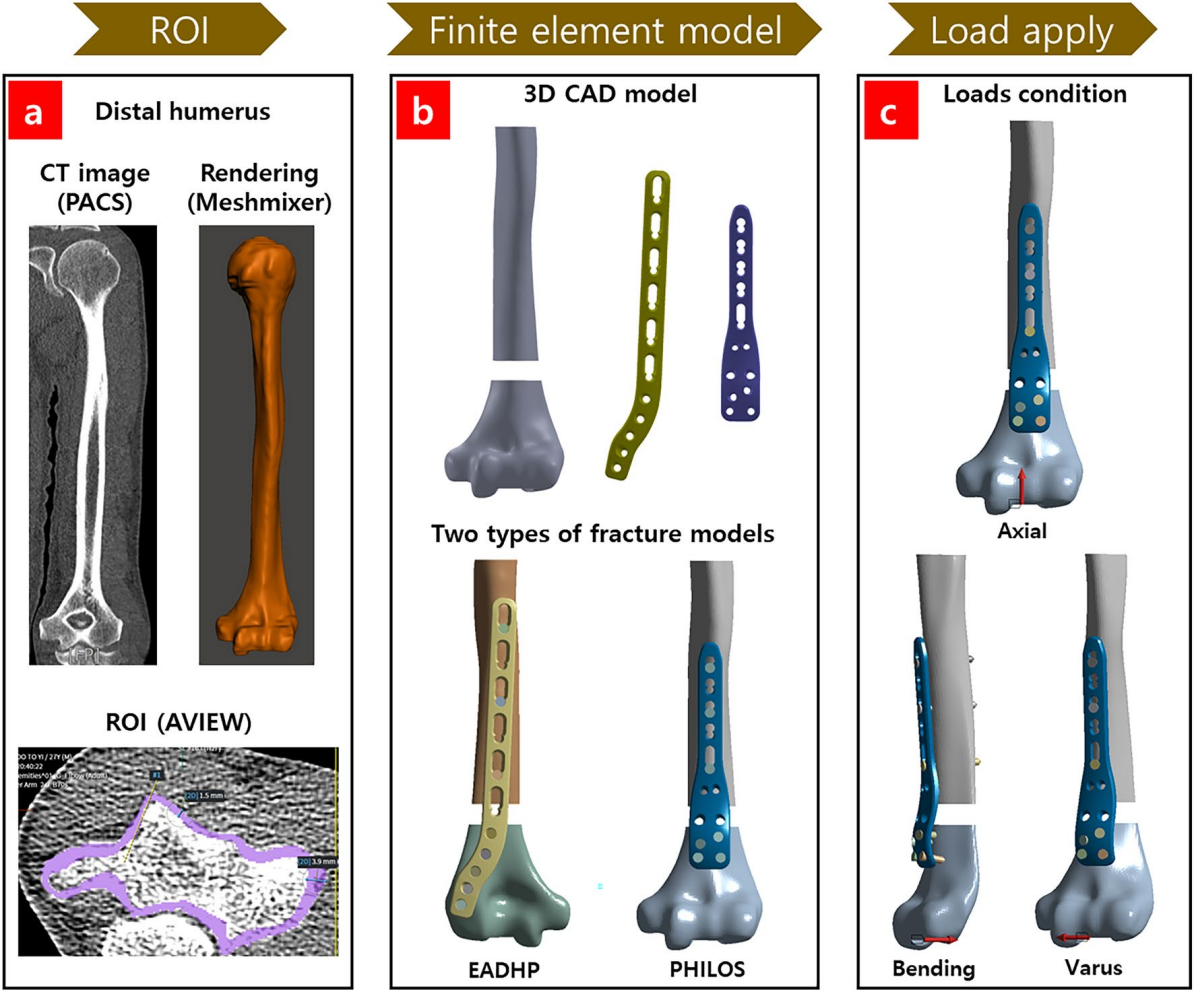
(**a**) Overall preprocessing; 3D reconstruction of distal humerus. Image review platform (AVIEW Research Build 1.1.42.12, Coreline Soft Inc., https://www.corelinesoft.com/research) was used for the region of interest (ROI) about the shape of the cortical bone. Also, Meshmixer ver. 3.5 (Autodesk, Inc., https://meshmixer.softonic.kr) program was used for smoothing and rendering. (**b**) 3D CAD model of 10 mm gap, distal humerus shaft fracture (DHSF), extra-articular distal humerus plate (EADHP), proximal humerus locking plate (PHILOS), and details on finite element models. (**c**) Three types of applied loads. Ansys Workbench 2022 R2 (ANSYS, Inc., Canonsburg, PA, USA, https://www.ansys.com) used for 3D modeling and of pre-process of finite element analysis (FEA).

The DHSF model for the FEA simulation was created by preprocessing computed tomography (CT) data, as shown in Fig. 1a.

First, the CT data of 10 adults without osteoporosis at Gachon University Gil Medical Center (IRB No. GDIRB2018-371) were acquired from the Picture Archiving and Communication System (PACS) to reconstruct the distal humerus into a 3D model, and then saved in the Digital Imaging and Communications (DICOM) format, which is the standard for medical images in PACS. The DICOM file was analyzed using an image review platform (AVIEW Research, Coreline Soft Inc.) for the segmentation of the region of interest (ROI) about the shape of the cortical bone. In CT image slice, visually check the outer and inner boundaries of the cortical bone, draw lines manually to perform the segmentation process of the ROI, and repeat this process for all slices. ROI of cortical bone was transformed into the NifTI format and saved in the STL format using the open-source medical imaging platform 3D Slicer program. Finally, the 3D model in STL format was smoothed and rendered using the Meshmixer (Autodesk, Inc.) program. This process was repeated to create 10 3D cortical bone 3D models from the CT data of 10 adults. Considering the shape and size of the distal humerus, cortical bone thickness, and attachment of the EADHP and PHILOS, one distal humerus 3D model was selected for the FEA simulation. The 3D model was based on the CT data of a 61-year-old male. The Hounsfield units used to identify the bones ranged from 300 to 1300. Continuous slice thickness (cSL) is 0.6 mm and total number of slices is 107, CT model is SOMATOM Definition Flash. In addition, CT image used post-processing technique of Freehand Slice (FFS). All methods were performed in accordance with the relevant guidelines and regulations. The informed consent was exempted by the Committee of Human Subjects Protection of the Gachon University Gil Medical Center and the Institutional Review Board (IRB) of Gachon University Gil Medical Center, South Korea (IRB No. GDIRB2018-371).

In contrast, the 3D models of the EADHP, PILOSH, and locking screws were made by measuring the dimensions of the real samples (Fig. 1b) provided by the manufacturer. The specifications of the EADHP, PHILOS, and locking screws are listed in Table 1.

**Table 1:** Details of the components of the EADHP and PHILOS used in the simulation.

Fracture models	Plate		Locking screw Ø3.5 mm (quantity)			
	Model	Length (mm)	Length = 25 mm	Length = 30 mm	Length = 35 mm	Length = 40 mm
EADHP	LCP distal humeral plate, extraarticular, 6 holes	158	1	4	2	1
PHILOS	Proximal humeral plate 3.5, 5 holes	114	1	6	–	–

The data preparation process is shown in Fig. 1a,b.

### Finite element model

The DHSF model was made by creating a 10 mm gap in the distal humerus 3D model, located 30 mm above the olecranon fossa^14^, as shown in Fig. 1b. Therefore, the 30 mm space was where the plate was attached from just above the olecranon fossa to the lower part of the fracture. It was determined that if the space is larger than 30 mm, then anterior plating can be performed; if the space is smaller than 30 mm, then posterior plating is performed. Therefore, it is clinically difficult to choose between anterior and posterior plating. In the 30 mm space, at least four locking screws can be used to perform distal fixation on the anterior plating with the PHILOS. The 10 mm gap was aimed at causing extreme instability in a biomechanical DHSF model^14,18^.

Two types of finite element models were developed using posterior and anterior plating with the EADHP and modified PHILOS, respectively, as shown in Fig. 1b.

The finite element models were meshed with 10 nodes of quadratic tetrahedral element with three degrees-of-freedom (DOF) for discretization^14^, mesh convergence study was performed and the element sizes of humerus, EADHP plate, PHILOS plate, and locking screws were 2.5 mm, 2.5 mm, 2.5 mm, and 1 mm, respectively. The total nodes and elements were: 24,903 and 91,961 (humerus), 5454 and 20,053 (EADHP plate), 4197 and 14,482 (PHILOS plate), 2224 and 6813 (locking screws in EADHP), 1807 and 5517 (locking screws in PHILOS).

### Material properties

The materials used in finite element models were assumed to be isotropic, homogeneous, and linear elastic. The mechanical properties of the cortical bone of the humerus^24,25^, EADHP, PHILOS and locking screws^26^ are listed in Table 2.

**Table 2:** Material properties assigned to individual the finite element models.

Property	Unit	Humerus	Locking plate system		
		Cortical bone	EADHP	PHILOS	Locking screw
Material		Normal bone	TiCP (grade 4)		Ti-6Al-7Nb
Young’s modulus	GPa	16	103		105
Poisson’s ratio		0.3	0.3		0.3
Ultimate tensile strength	MPa	146	550		900

### Boundary condition

With regard to the contact conditions at the component interface, it was assumed that the plate and locking screws, as well as the locking screws and cortical bone, were completely bonded. The pre-load of the locking screws was not applied. A friction coefficient of 0.3 was applied to allow finite sliding^14^ between the cortical bone and plates should contact occur owing to the deformation under load conditions.

Three types of loads condition were applied to the elbow joint under the support condition, in which the end of the humerus shaft was fixed, as shown in Fig. 1c. The applied loads were within the physiological limits of loads in daily activities during the postoperative rehabilitation period^14,27^. These include an axial compression force of 200 N applied vertically on the elbow joint; a bending force of 30 N^14,27^; and a varus force of 30 N, which is the gravity acting on the forearm while the elbow is flexed and extended, with pronounced clinical significance^14,27^.

### Institutional review board statement

This study was approved by the Institutional Review Board (IRB) of Gachon University Gil Medical Center, and the protocols used in the study were approved by the Committee of Human Subjects Protection of the Gachon University Gil Medical Center, South Korea (IRB No. GDIRB2018-371). The study was approved by the Institutional Review Board (IRB) of Gachon University Gil Medical Center. The protocols used in the study were approved by the Hospital’s Protection of Human Subjects Committee.

## Results

FEA simulations were performed to quantitatively compare and evaluate the biomechanical performance between posterior plating with the EADHP and anterior plating with the modified PHILOS under the three load conditions. Six simulations were conducted, yielding 18 values, used Ansys Workbench 2022 R2 (ANSYS, Inc., Canonsburg, PA, USA) computer aided engineering (CAE) software. Non-linear analysis was applied to the contact surface to which the friction coefficient was applied, and linear analysis was applied to other regions.

This study aims to determine the fixation range and state of anterior plating with the modified PHILOS. We focused on the stress distribution of the EADHP and PHILOS. Thus, we attempt to consider the mechanical properties of the material from the perspective of mechanical analysis.

The simulation results of the EADHP and PHILOS are summarized in Table 3. As in a previous biomechanical study using cadavers^18^, the EADHP and PHILOS exhibited adequate biomechanical performance and stability under axial, bending and varus load conditions. The maximum von-Mises stress of the EADHP on the plate was 300.54 MPa under the axial force, while those of the PHILOS were 109.62 MPa and 83.63 MPa on the humerus under the bending and varus forces, respectively.

**Table 3:** Comparison of maximum von-Mises stress on posterior plating with the EADHP and anterior plating with the modified PHILOS.

Load condition		Fracture model	Maximum von-Mises stress (MPa)		
Type	Magnitude		Humerus	Plate	Locking screws
Axial force	200 N	EADHP	17.10	300.54	37.14
		PHILOS	10.97	57.55	16.82
Bending force	30 N	EADHP	8.08	137.41	14.21
		PHILOS	109.62	128.66	22.13
Varus Force	30 N	EADHP	6.38	130.84	16.96
		PHILOS	83.63	98.79	13.32

**Figure 2:**
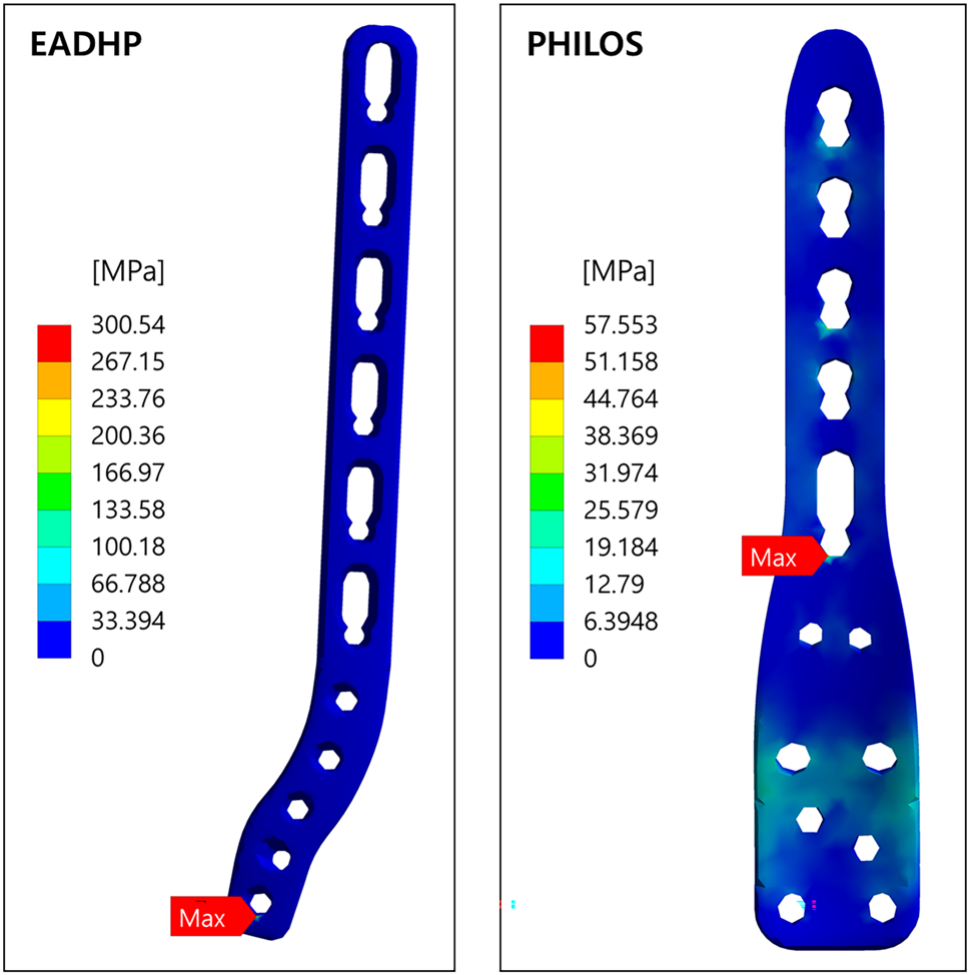
Maximum von-Mises stress on the plate under axial force condition. Ansys Workbench 2022 R2 (ANSYS, Inc., Canonsburg, PA, USA, https://www.ansys.com) used for finite element analysis (FEA) simulations.

With regard to the stress concentrations in the EADHP under the axial force, the maximum von-Mises stress of 300.54 MPa occurred at the edge of a locking screw hole near the lateral epicondyle on the plate, as shown in Fig. 2. However, the amount of stress remained below the ultimate tensile strength (UTS) of 550 MPa. The PHILOS had a maximum von-Mises stress of 57.55 MPa on the locking screw hole of the humerus shaft and the plate fixed just above the fracture.

**Figure 3:**
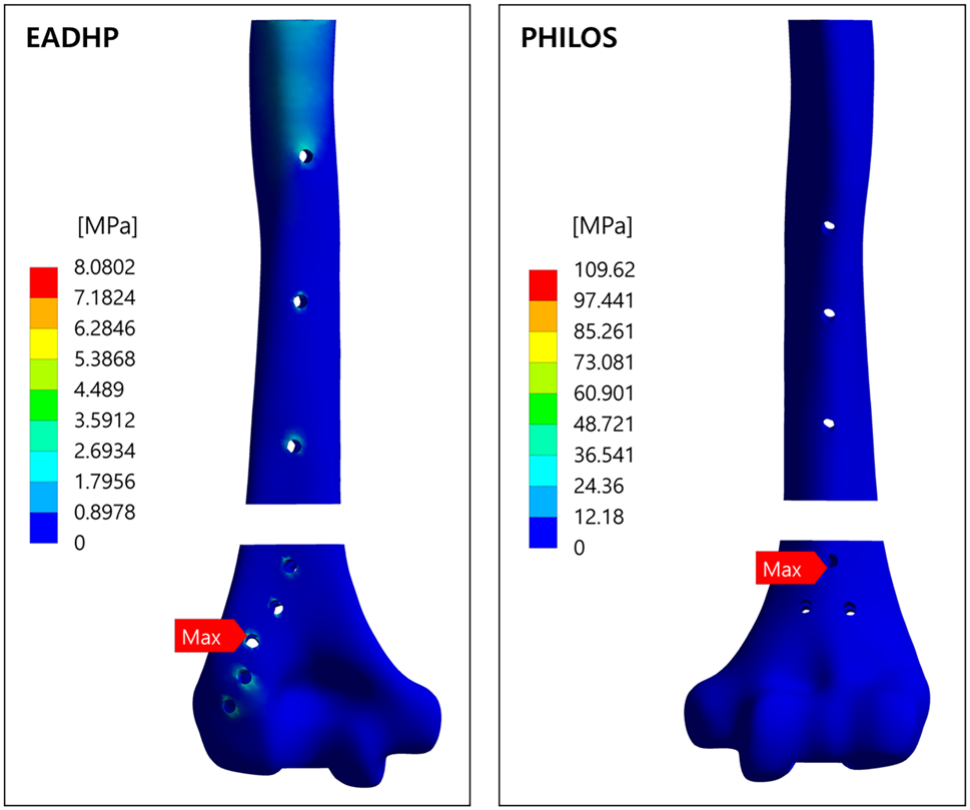
Maximum von-Mises stress on the humerus under bending force condition. Ansys Workbench 2022 R2 (ANSYS, Inc., Canonsburg, PA, USA, https://www.ansys.com) used for finite element analysis (FEA) simulations.

Figure 3 shows the simulation results under the bending force condition. The PHILOS showed a maximum von-Mises stress of 109.62 MPa at the edge of a locking screw hole located in the middle of the lateral epicondyle and lateral supracondylar ridged on the humerus, as indicated by the red arrow, owing to stress concentration. This value is approximately 13 times higher than that of the EADHP but was stable because it is less than the UTS of 146 MPa. However, because of its proximity to the UTS, it cannot be concluded that the fixed stability is always secured even with changes in other conditions. Even under varus force conditions, the maximum von-Mises stress in the PHILOS on the humerus was 83.63 MPa, which is approximately 13 times higher than that of the EADHP of 6.38 MPa.

**Figure 4:**
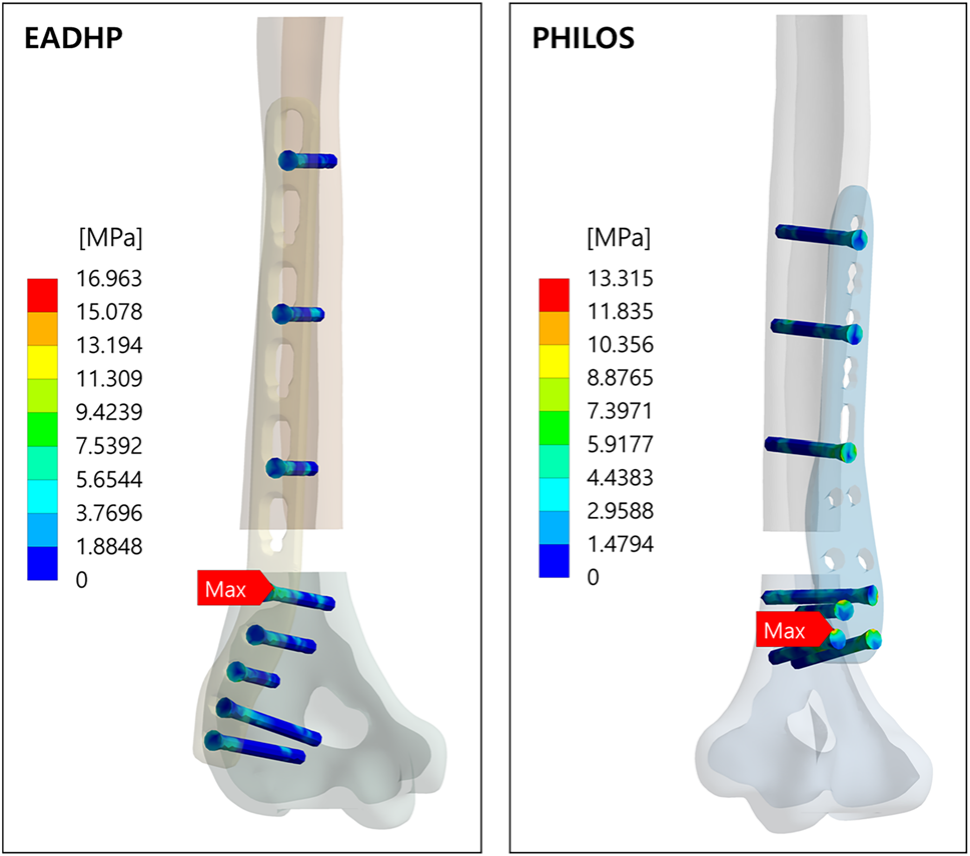
Maximum von-Mises stress on the locking screws under varus force condition. Ansys Workbench 2022 R2 (ANSYS, Inc., Canonsburg, PA, USA, https://www.ansys.com) used for finite element analysis (FEA) simulations.

The maximum von-Mises stress of the plate and locking screws in the EADHP and PHILOS under bending and varus force conditions had comparable values. The locking screws had a maximum von-Mises stress of 16.96 MPa and 13.32 MPa on the head under varus force conditions, as shown in Fig. 4, confirming the adequate distribution of the external force within the safe range.

## Discussion

FEA was conducted to quantitatively compare and evaluate the biomechanical performance between posterior plating with the EADHP, which is representative fixation method in DHFS; and anterior plating with a modified (upside-down application) PHILOS, which is the proposed alternative method. To cause extreme instability in the biomechanical DHSF model, a fracture with a 10 mm gap was applied, and the stress distribution was analyzed, focusing on the case of applying the loads of daily activities during the postoperative rehabilitation period.

Visual and quantitative evidence were provided to predict the fixation state of the EADHP and PHILOS. The biomechanical structures of distal humerus were created virtually using CT data and the 3D model of the distal humerus was reconstructed. The physical properties of the distal humerus were also investigated. The reproducibility of the results was ensured by providing the details on reconstructing the 3D models from raw data and material information of the DHSF model, EADHP, and PHILOS.

This study has two main contributions. One is the implementation of two types of finite element models (posterior plating with the EADHP and anterior plating with the modified PHILOS) that represent the fixation methods in DHFS. The other is the results of the FEA performed to quantitatively compare and evaluate the biomechanical performance between posterior plating with the EADHP and anterior plating with the modified PHILOS. As a result, we determined the fixation range and state of the PHILOS. In previous studies, anterior plating was performed with the modified PHILOS^22,28^, and a comparison was made between posterior plating with the EADHP and anterior plating with the modified PHILOS^6^. Biomechanical study using cadavers to compare posterior plating with the EADHP and anterior plating with the modified PHILOS have also been conducted^18^. However, to the best of our knowledge, there has been no FEA study that quantitatively compared and evaluated the biomechanical performance between posterior plating with the EADHP and anterior plating with the modified PHILOS.

Under the three applied load conditions, the biomechanical performance of the fixation treatments was quantitatively compared and evaluated by analyzing the stress concentration and contribution in a stress contour map, which visualized the stress gradient and identified the maximum stress. The fixation failure, i.e. fixed stability, was predicted by defining breakage as an excess of the UTS.

The simulation results showed that the EADHP and PHILOS had adequate biomechanical performance and stability under axial, bending, and varus load conditions. In particular, under bending and varus force loads, the EADHP properly distributed the stress in the humerus to the plate. The PHILOS realized the purpose of fixation, even though stress concentration occurred in the humerus. The PHILOS achieved the required fixation using only four locking screws within a fixation range of 30 mm just above the olecranon fossa.

Under the axial force condition, the EADHP and PHILOS showed adequate stress contributions on the humerus and locking screws. However, as shown in Fig. 2, the EADHP and PHILOS had a maximum stress of 300.54 MPa and 57.55 MPa on plate owing to stress concentration, respectively. The fixed stability was ensured because it was less than the UTS of 550 MPa. All maximum stresses occurred at the edge of the locking screw hole, which was attributed to the increased local pressure on the contact surface between the plate and locking screws. Overall, the PHILOS exhibited a better stress distribution compared with that of the EADHP. As shown in Fig. 1b, the EADHP has an asymmetrical shape, is longer (158 mm) than the PHILOS (114 mm), narrower, and thicker. The cross-sectional areas of the EADHP and PHILOS plates located at site of the 10 mm gap are like 57.48 mm^2^ and 55.2 mm^2^, respectively. However, the asymmetrical shape of the EADHP caused an additional bending moment under the axial force. As shown in Fig. 2, stress concentration occurred at the furthest point from the humerus shaft, which is the fixed part, i.e. at the position where the bending moment occurred the greatest. This confirms that the performance of the PHILOS is better than that of the EADHP under axial force conditions owing to its symmetrical shape.

The EADHP was expected to have a better stress distribution compared with the PHILOS under bending and varus forces conditions because it can be attached to the coronoid fossa side owing to the “J”-shaped plate precontoured design^16–20^ and length is long. In simulation of the bending force, the maximum von-Mises stress of the PHILOS on the humerus was 109.62 MPa, which is approximately 13 times higher than that of the EADHP of 8.08 MPa, as shown in Fig. 3. Similarly, the maximum von-Mises stress of the PHILOS was 83.63 MPa, which is approximately 13 times higher than that of the EADHP of 6.38 MPa under the varus force. The results of the bending and varus force simulations on the plate showed that stress of the EADHP of 137.41 MPa and 130.84 MPa was higher than those of PHILOS of 128.66 MPa and 98.79 MPa, respectively. The structural stability was secured by considering the UTS of 550 MPa. The maximum von-Mises stress of the EADHP and PHILOS on the locking screws was 30 MPa or less. This confirmed a high fixed stability considering the UTS of 900 MPa. As expected, the EADHP showed better stress distribution than the PHILOS under bending and varus force conditions. Therefore, both the EADHP and PHILOS showed adequate biomechanical performance and stability under bending and varus force conditions.

More specifically, the comparative analysis results of the FEA simulations of the EADHP and PHILOS showed the following results. First, the EADHP properly distributed the stress on the humerus to the plate under bending and varus force conditions. As a result, the humerus was subjected to a maximum stress of 10 MPa or less, while the maximum stress on the plate was 140 MPa or less. The EADHP also distributed stress more effectively than the PHILOS. The attachment range of the EADHP is the arrangement in which a total of five locking screws were fixed in a row from the coronoid fossa side to the lower part of the 10 mm fracture of the humerus. In contrast, the PHILOS was originally designed for proximal humerus fractures^18,22^. A modified PHILOS was applied in which the upper part attached to the proximal humerus was attached to the lower part in the direction of an elbow joint. Therefore, the attachment range of the PHILOS was limited to 30 mm from just above the olecranon fossa to the lower part of the fracture, and only four locking screws were used. For this reason, the PHILOS ineffectively distributed the stress under bending and varus force conditions. The maximum von-Mises stress equivalent to 75% of the UTS of 146 MPa occurred on the humerus.

The PHILOS was structurally stable but did not exhibit good stress distribution under bending and varus force conditions. As mentioned above, the maximum von-Mises stress equivalent to 75% of the UTS resulted from constraints on the symmetrical shape and a fixation range of 30 mm. Because the maximum stress was less than the UTS, the PHILOS could be considered as having achieved the purpose of fixation despite the stress concentration on the humerus. However, the stress distribution on the humerus must be considered because the maximum stress on the humerus under the bending force condition is close to the UTS. If the thickness of the cortical bone of the humerus decreases, then the maximum stress on the humerus will increase. If the osteoporotic humerus is affected, then the Young’s modulus and UTS will decrease. As the maximum stress on the humerus increased, the Young's modulus and stiffness decreased, the UTS (i.e. breakage criterion) decreased, and the possibility that the maximum stress will exceed the UTS increased. The size, bone thickness, and bone density of the humerus affect its strength and stiffness, and eventually determine the magnitude of stress that could be loaded. Therefore, in an actual clinical environment, a sufficient review of the thickness and bone density of the humerus is required along with anatomical conditions, such as the size and shape of the humerus. A quantitative comparison of the correlation between bone thickness and fixation strength, and the biomechanical performance between normal bone and osteoporotic humerus will be an interesting topic of study.

In conclusion, this comparative study showed that anterior plating with the modified (upside-down application) PHILOS exhibited a biomechanical performance comparable to that of posterior plating with the EADHP. From the perspective of minimally invasive surgery (MIS), the EADHP is fixed with five locking screws from the coronoid fossa side to 30 mm just above the olecranon fossa; is longer (158 mm) than the PHILOS (114 mm); and has a volume of 6723 mm^3^ (which is 1.84 times higher than that of the PHILOS of 3646 mm^3^). Therefore, the PHILOS could be an alternative for the fixation of a DHSF when considering the dissection range and complaints (e.g. skin irritation) associated with the EADHP^6,10,18,19,22,23,29^.

In line with our findings, retrospective studies of the PHILOS alone or in comparison with the EADHP have shown satisfactory clinical and radiologic outcomes^6,22,28^. In a biomechanical study using cadavers, Lim et al.^18^ reported that anterior plating with a modified PHILOS showed a fixation and biomechanical performance comparable to those of posterior plating with the EADHP. This cadaveric study differs from our study because the biomechanical performance was evaluated by measuring stiffness at the time of breakage. Because the stiffness has no maximum value such as the UTS, it can be compared under conditions that consider the application environment. The reason is that when the stiffness becomes too high, the structure becomes rigid; hence, the high stiffness is not always advantageous. Another difference is that no research has been conducted on the fixation range of the PHILOS and the number of screws that should be fixed.

This study has several limitations. First, in the process of reconstructing the distal humerus as a 3D model, 10 cortical bone 3D models were created from the CT data of 10 adults. One distal humerus 3D model was selected by considering the shape and size of the humerus, cortical bone thickness, and attachment of the EADHP and PHILOS. Considering the statistical generality of the results, it was essential to acquire multiple datasets categorized by sex and age to determine the shape and thickness of the humerus. Second, for convenience of analysis and cost reduction, the humerus was reconstructed as a cortical bone, simplified finite element models were considered, and complicated anatomical structures (e.g. muscles and ligaments), were excluded. Although the humerus was modeled only as a cortical bone, it is expected to have a small effect on the FEA results. The reason is that the cortical bone is assembled with locking screws back and forth, and the Young’s modulus of the cortical bone is 16 GPa, which is 29 times larger than 0.55 GPa of the trabecular bone^25^, therefore, even if there is a trabecular bone, as a result, most of the stress on the humerus was expected to occur in the cortical bone. Third, the three types of load conditions applied in the simulation were designed by considering the clinical and rehabilitation environments, and the commonly adopted values in previous studies. Further research is required on various clinical and rehabilitation environments. Fourth, we assumed the humerus as an isotropic material and defined breakage as an excess of the UTS. Thus, the assumption of linear material properties would be invalidated in cases where the maximum stress exceeds the YS, even if it does not exceed the UTS. Finally, it is necessary to validate the finite element model by comparing the results of biomechanical studies performed using the cadaveric distal humerus.

Despite these limitations, to the best of our knowledge, this is the first FEA study that quantitatively compared and evaluated the biomechanical performance between posterior plating with the EADHP and anterior plating with the modified PHILOS. The study determined the fixation range and state of anterior plating with the modified PHILOS in DHFS through FEA.

## Conclusion

The FEA simulation results of both posterior plating with the EADHP and anterior plating with the modified (upside-down application) PHILOS showed adequate biomechanical performance and stability under axial, bending, and varus load conditions. It was confirmed that the PHILOS has a fixed stability comparable to that of the EADHP. The PHILOS achieved the required fixation effect using only four locking screws within a fixed range of 30 mm from just above the olecranon fossa. Therefore, the PHILOS could be an alternative for the fixation of DHSF when considering the dissection range and complaints (e.g. skin irritation) associated with the EADHP.

## Data Availability

All data used to support the findings of this study are available upon request from the corresponding author.
